# Visual effects and rehabilitation after stroke

**Published:** 2017-03-03

**Authors:** Fiona Rowe

**Affiliations:** 1 Reader in Orthoptics and Health Services Research, Hon Professor of Orthoptics UTS, University of Liverpool, Liverpool, UK. **www.liverpool.ac.uk/psychology-health-and-society/research/vision/about**

**Strokes, or cerebrovascular accidents (CVA) are common, particularly in older people. The problems of motor function and speech are well known. This article explains the common visual problems which can occur with a stroke and gives information about diagnosis and management.**

**Figure:**
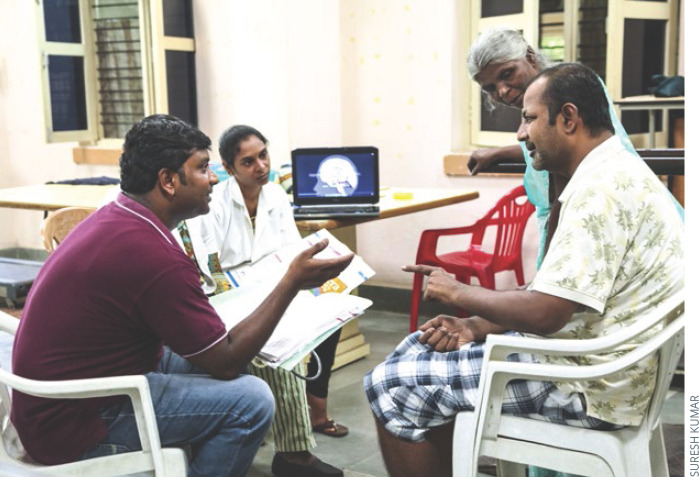
A patient receiving information on rehabilitation. INDIA

## What is a stroke?

A stroke occurs when there is an interruption to blood flow to the brain either because of a blood clot blocking the blood vessel or a haemorrhage in the brain^1^. Strokes can cause signs which are obvious, such as loss of speech, drooping of one side of their face, or weakness or paralysis of the arm and/or leg on one side of the body^1^. The vision is affected in about two thirds of people who have had a stroke, but this is often not obvious to the patient or their carers. For example, someone who has weakness down one side may bump into things or not eat all the food on their plate, not realising that this may also be because they have visual field loss^2^.

## What causes a stroke?

A stroke or cerebrovascular accident, (CVA) is the result of a blocked blood vessel in the brain (thrombosis or embolus), or haemorrhage into the brain^1^. Strokes are more likely in the elderly, and those who have high blood pressure, diabetes or cardiovascular disease.

## Types of visual loss in people who have had a stroke

There are four ways in which vision can be affected following a stroke:

1. Loss of central vision
2. Visual field loss
3. Visual perceptual abnormalities
4. Eye movement abnormalities

**Figure 1:**
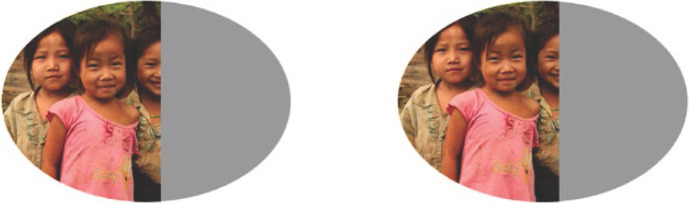
Right homonymous hemianopia: the right-hand field of view is lost in both eyes

These may occur in isolation but more frequently occur in combination^3^.

Problems with **central vision** are quite common after a stroke. The symptoms include blurred or altered vision. In many the vision improves, but the visual loss can be permanent.

**Figure 2:**
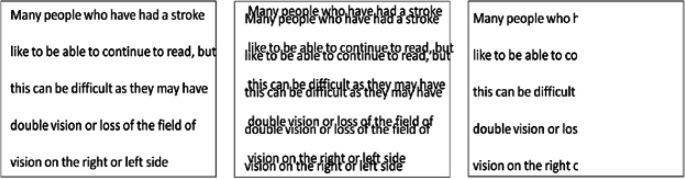
Impact of vertical double vision (central image) and right hemianopia (right image) on reading

**Visual field loss** occurs in up to half of people with a stroke, with the commonest defect being homonymous hemianopia in which vision is lost in the right or the left visual fields^4^ (**Figure 1**). Patients may not be aware of this, and bump into doorframes or trip over things on the affected side. Reading can also be difficult (**Figure 2** on page 74).

**Visual perceptual deficits** are many and varied affecting about a third of people with a stroke. Problems that may develop include neglect one side of their body; difficulty recognising faces or objects, or difficulties with colour vision, depth perception and motion^5^.

**Eye movement abnormalities** can also be varied, including strabismus (misaligned eyes), difficulty in converging the eyes to look at near objects, or double vision due to the cranial nerves which control eye movement being affected^6^. Typical symptoms include double vision, or jumbled, blurred and/or juddery vision (**Figure 2** on page 74).

## Impact

Blurred vision, double vision and loss of visual field are significant symptoms that impair daily functioning^7^.

The patient or their close relatives may report that they frequently bump into objects such as door frames; have difficulty finding things on surfaces; are unsure of their footing while walking and stumble; may leave food uneaten on one side of the plate and have difficulty with reading. Other impacts on the quality of life include loss of confidence, fear of falling, fear of going out alone, social isolation and loss of independence^8^.

## How to assess visual function in someone who has had a stroke

Examination for visual loss^9^ is essential for stroke survivors. There are various assessment tools which can be used to examine visual function after a stroke:

- UK National Clinical Guidelines for Stroke: **www.rcplondon.ac.uk/guidelines-policy/stroke-guidelines**
- UK stroke/vision resources and factsheets: **www.parallelvisionproblems.org.uk/index.php/eye-conditions/**
- UK Stroke Association stroke/vision factsheet: **www.stroke.org.uk/what-stroke/common-problems-after-stroke/vision-problems**
- UK Royal National Institute for the Blind stroke/vision factsheet: **www.rnib.org.uk/eye-health-eye-conditions-z-eye-conditions/stroke-related-eye-conditions**)

## Management

Treatment options aim to restore visual function to as normal as possible^10^. For eye movement abnormalities, prisms and patching one eye can be effective in reducing double vision^6^. For visual field loss a Cochrane systematic review^11^ reports favourable evidence of visual scanning training which aims to compensate for the visual field loss. It is available as a paper training option (**www.strokevision.org.uk**) or through computer training (**www.eyesearch.ucl.ac.uk; www.readright.ucl.ac.uk**).

Stroke survivors with persistent impairment of central vision may be helped by low vision services which can include magnifiers, reading aids, computerised adaptations and improved lighting^12^. Furthermore, simple adaptations can be made by stroke survivors such as using large print, ensuring good lighting at home, putting labels or coloured stickers on cooking equipment, decluttering areas and having a companion when going out, particularly in busy, crowded places^10^.

## Conclusion

Post-stroke difficulties in visual function are an under-recognised problem that cause significant impact to the quality of life of stroke survivors. Carers and health workers need to be aware that problems with vision are a common consequence of stroke that is not outwardly obvious.

Assessment including visual functioning is best provided as part of a multi-disciplinary team on acute stroke units, or in neuro-rehabilitation units. A careful history about visual problems from the patient and carers followed by examination of visual acuity, eye movements and visual field are important in understanding the difficulties in visual functioning.

Management should be tailored to each individual, their visual difficulties and visual needs. With about one quarter of stroke survivors being of working age, rehabilitation in the conext of adaptation of the work place environment is vital if younger people are to return to work after stroke. Rehabilitation requires patience and perseverance on the side of the client, relatives and the health provider.

Despite improvement in stroke prevention and acute stroke management, the increasing ageing population will result in more stroke survivors requiring rehabilitation. Policy makers need to understand the Importance of providing post-stroke rehabilitation services including visual functioning.

## References

[1] World Health Organization. Stroke and cerebrovascular accident. 2017. **http://www.who.int/topics/cerebrovascular_accident/en/**

[2] Hepworth LR Rowe FJ Walker MF Rockliffe J Noonan C Howard C Currie J. Post-stroke Visual Impairment: A Systematic Literature Review of Types and Recovery of Visual Conditions. Ophthalmology Research: An International Journal. 2015; 5(1). ISSN: 2321-7227

[3] Rowe FJ, VIS group. Visual impairment following stroke. Do stroke patients require vision assessment? Age and Ageing. 2009; 38: 188–193 19029069 10.1093/ageing/afn230

[4] Rowe FJ, VIS UK. A Prospective Profile of Visual Field Loss following Stroke: Prevalence, Type, Rehabilitation, and Outcome. BioMed Research International, vol. 2013, Article ID 719096, 1–12, 2013. doi:10.1155/2013/719096. 10.1155/2013/719096 PMC3782154 24089687

[5] Rowe FJ, VIS group. Visual perceptual consequences of stroke. Strabismus 2009; 17: 24–28 19301189 10.1080/09273970802678537

[6] Rowe FJ, VIS group. The profile of strabismus in stroke survivors. Eye 2010; 24: 682–5 19521433 10.1038/eye.2009.138

[7] Rowe FJ, VIS Group. Symptoms of stroke related visual impairment. Strabismus, 2013; 21: 150–4 23713941 10.3109/09273972.2013.786742

[8] Hepworth L Rowe FJ. Visual impairment following stroke – the impact on quality of life: a systematic review. Ophthalmology Research: an international journal. 2016; 5(2): 1–15

[9] Rowe FJ. The importance of accurate visual assessment after stroke: Editorial. Expert Reviews in Ophthalmology. 2011: 6; 133–6

[10] Rowe FJ. Care provision and unmet need for post stroke visual impairment; Final report. 2013. **www.stroke.org.uk/sites/default/files/final_report_unmet_need_2013.pdf?**

[11] Pollock A Hazelton C Henderson CA Angilley J Dhillon B Langhorne P Livingstone K Munro FA Orr H Rowe FJ Shahani U. Interventions for visual field defects in patients with stroke. Cochrane Database of Systematic Reviews 2011, Issue 10. Art. No.: CD008388. DOI: 10.1002/14651858.CD008388.pub2. 10.1002/14651858.CD008388.pub2 21975779

[12] Virgili G Rubin G. Orientation and mobility training for adults with low vision. Cochrane Database of Systematic Reviews 2010, Issue 5. Art. No.: CD003925. DOI: 10.1002/14651858.CD003925.pub3 10.1002/14651858.CD003925.pub3 PMC7138242 20464725

